# A retrospective study of breast carcinoma: causes of death and pattern of metastases.

**DOI:** 10.1038/bjc.1989.292

**Published:** 1989-09

**Authors:** D. M. Parham, A. J. Robertson

**Affiliations:** Department of Pathology, Ninewells Hospital and Medical School, University of Dundee, UK.

## Abstract

Autopsy reports and clinical request forms between March 1973 and September 1986 were reviewed for patients with a clinical history of breast carcinoma. During this period 85 cases were identified. The causes of death and metastatic pattern of the breast carcinoma were recorded. Only 25 (29%) of these patients died as a direct consequence of this disease. Of the 85 cases in total, 28 were thought clinically to have died as a result of breast carcinoma but autopsy confirmed these findings in only 21 (75%).


*Br. J. Cancer* (1989), **60**, 394–396 © The Macmillan Press Ltd., 1989

### A retrospective study of breast carcinoma: causes of death and pattern of metastases

D.M. Parham & A.J. Robertson

*Department of Pathology, Ninewells Hospital and Medical School, University of Dundee, Dundee DD1 9SY, UK.*

**Summary** Autopsy reports and clinical request forms between March 1973 and September 1986 were reviewed for patients with a clinical history of breast carcinoma. During this period 85 cases were identified. The causes of death and metastatic pattern of the breast carcinoma were recorded. Only 25 (29%) of these patients died as a direct consequence of this disease. Of the 85 cases in total, 28 were thought clinically to have died as a result of breast carcinoma but autopsy confirmed these findings in only 21 (75%).

Breast carcinoma is reported to be the most common fatal malignant disease among women in the Western world (Mould, 1983) yet much is still unknown about its natural history. Considerable effort has been directed towards improving diagnosis and treatment but only scant attention has been paid to the actual cause of death in patients with this disease. Since 1950, in the English language literature, only a few autopsy studies, all from American hospitals, have been reported (Abrams *et al.*, 1950; Meissner & Warren, 1971; Sproul, 1971; Viadana *et al.*, 1973; Cifuentes & Pickren, 1979; Cho & Choi, 1980; Hagemeister *et al.*, 1980; Amer, 1982). It is obviously important that data on cause of death and presence of residual disease should be collected to enable realistic comparison of the efficacy of different modalities of treatment and accurate calculation of survival times. It was therefore decided to evaluate the causes of death and ascertain the distribution of residual disease in breast carcinoma patients autopsied in this hospital.

### Materials and methods

All autopsy records with clinical summaries between March 1973 and September 1986 were reviewed for female patients with a previous history of breast carcinoma. A total of 85 cases diagnosed and treated before their last admission were identified. Information regarding histological sub-type of the neoplasm, non-surgical treatment modalities or menstrual status was not generally available. Cases where breast cancer was diagnosed on the patient's last admission or was an incidental autopsy finding were excluded. A further eight women who had previously had mastectomies were excluded from the study as it was not possible to be certain about the underlying pathology from the records available. None of these excluded cases had evidence of metastatic breast disease at autopsy.

The clinical summary accompanying the autopsy request form and the autopsy findings were considered together to determine the cause of death in each case. A case was deemed a breast carcinoma death in the following circumstances: (1) when extensive metastases were present in the brain, liver, lung, heart or involved other vital structures; (2) when the carcinoma was the cause of infection or sepsis; (3) when the carcinoma was the source of significant haemorrhage; or (4) when metastatic disease had given rise to a pulmonary embolus as a result of immobility and deep venous thrombosis. A non-breast carcinoma death was due to causes unrelated to breast carcinoma and in the absence of significant metastatic breast disease. The finding of occasional small secondary lesions within, for example, the liver or a lymph node was considered insignificant to the cause of death if this was in agreement with autopsy findings and clinical summaries. Examples of non-breast carcinoma deaths included myocardial infarction or second primary malignancies.

All the autopsies were carried out at Ninewells Hospital and Medical School. In two autopsies the brain was not removed but these were included in the series as metastatic brain disease was not suspected clinically. It is usual practice in this hospital to sample for histology any macroscopic abnormality and to take a single tissue block from all major organs. Histology was available in all but two cases. Neither of these cases had gross evidence of metaststic disease at post mortem.

### Results

A summary of patient characteristics is shown in Table I and survival curves are shown in Figure 1. A review of the autopsy and clinical findings indicated that only 25 (29%) of the 85 cases of previously treated breast carcinoma died as a consequence of the disease. The length of survival of these patients was significantly shorter (median 31 months as opposed to 115 months; Mann–Whitney U test, $P=0.008$) than that observed in non-breast cancer deaths. The terminal events in these 25 patients were bronchopneumonia (9 cases), carcinomatosis (7), pulmonary embolus (5), DIC (1), renal failure (1), respiratory obstruction (1) and a malignant pleural effusion (1). In three further cases breast carcinoma was thought to play a possible role in the patient's death. In the first case the possibility was raised that systemic hypertension which had resulted in a subarachnoid haemorrhage may have been due to secondary breast carcinoma causing ureteric obstruction. In the second case a subarachnoid haemorrhage had been precipitated by

**Table I** Population characteristics

| | |
|---|---|
| Period of study | March 1973 to September 1986 |
| Number of autopsied patients | 85 |
| Age at diagnosis | Mean 60 years (range 31–86) |
| *Survival* | |
| All patients | Median 90 months (range 0–416)<br>64% five-year survival |
| Breast carcinoma deaths | Median 31 months (25 cases) |
| Non-breast carcinoma deaths | Median 115 months (57 cases) |
| *Site of breast cancer* | |
| Left | 39 |
| Right | 44 |
| Bilateral | 2 |
| *Surgical treatment* | |
| Mastectomy | 69 |
| Bilateral mastectomy | 2 |
| Lumpectomy | 2 |
| Non-surgical treatment | 12 |

Correspondence: D.M. Parham.
Received 29 December 1988, and in revised form, 22 March 1989.

**Figure 1** Survival curves for (**a**) breast carcinoma deaths (25 cases), (**b**) all patients and (**c**) non-breast carcinoma deaths (57 cases).

thrombocytopenia as a result of a myeloproliferative disease which had developed after radiotherapy. In the third case death was a consequence of disseminated intravascular coagulation which may have been precipitated by either radionecrosis of the breast carcinoma or a recent myocardial infarction.

In 28 patients death was suspected clinically to be a definite consequence of breast carcinoma. This was correct in 21 (75%) cases with a further case where the breast cancer may have played a role in the patient's demise. In a further 12 patients the possibility of a breast cancer death was raised clinically. This was correct in one case, with a further case where breast carcinoma may have played a role in the patient's demise. The primary pathology in the cases suspected clinically to have died of breast carcinoma but who died from other causes is shown in Table II.

In 43 cases death was thought by the clinicians to have occurred independent of the patient's breast carcinoma – this was incorrect in two (5%) cases with a further case where the disease may indeed have played a role in the patient's death. In two cases no clinical opinion as to the cause of death was stated – in one of these death was a result of metastatic breast disease. Table III lists the cause of death in all the non-breast cancer deaths.

The incidence of metastases in the various organs of all 85 cases is shown in Table IV. In all cases with metastases the tumour was of infiltrating 'ductal' type (not otherwise specified). Of the 57 patients who died of causes unrelated to breast cancer 15 had evidence of residual disease. Nine of these had residual breast disease as a result of non-operative treatment. Three patients had recurrent disease at their mastectomy sites and sites of metastatic disease included regional lymph nodes (4 cases), liver (3 cases), lung (2 cases) and bone (1 case).

**Table II** Causes of death in group clinically suspected to be due to breast cancer but dying of other causes

| *Cause* | *Number* |
|---|---|
| Myocardial ischaemia | 4 |
| Bronchopneumonia | 4 |
| Ovarian carcinoma | 1 |
| Astrocytoma | 1 |
| Pneumoccocal meningitis | 1 |
| Cerebral haemorrhage | 1 |
| Small intestinal obstruction (Richter's) | 1 |
| Peptic ulcer (complications of) | 1 |
| Pulmonary embolus | 1 |

In one case cause of death was uncertain from clinical and autopsy findings.

**Table III** Major pathology causing death in patients not succumbing to breast carcinoma

| *Cause* | *Number* |
|---|---|
| *Cardiovascular* | |
| Myocardial ischaemia | 13 |
| Mitral valve disease (rheumatic) | |
| Congestive cardiac failure (aortic stenosis) | |
| Ruptured aortic aneurysm | 1 |
| *Respiratory* | |
| Bronchopneumonia (not otherwise specified) | |
| Aspergillous bronchopneumonia (bronchiectasis) | |
| Aspergilloma (PMH TB) | 1 |
| Acute bronchitis | 1 |
| Bronchial carcinoma | 3 |
| Carcinoma larynx | 1 |
| Chronic obstructive airways disease | |
| Pulmonary embolus | 4 |
| *Gastrointestinal* | |
| Pancreatic carcinoma | 1 |
| Colonic adenocarcinoma | 1 |
| Acute pancreatitis | 1 |
| Complications of peptic ulceration | |
| Small intestinal obstruction (Richter's hernia) | |
| *Genital urinary* | |
| Glomerulonephritis | 1 |
| Pyelonephritis | 2 |
| Adult polycystic kidney disease | |
| Ovarian carcinoma | 4 |
| *Nervous system* | |
| Pneumococcal meningitis | 2 |
| Astrocytoma | 1 |
| Cerebral haemorrhage | 2 |
| *Other* | |
| Hodgkin's disease | 1 |
| Acute myeloid leukaemia | 1 |
| Myeloma | 1 |

In one case cause of death was not clear from either autopsy or clinical summary.

### Discussion

The reliability of death certification based on clinical findings is doubtful (Editorial, 1966). In a prospective study based on 1,152 deaths from all causes, Cameron & McGoogan (1981) were only able to confirm the major cause of death clinically in 61% of autopsies. The findings in this study of patients with treated breast cancer are similar, in that where a confident clinical diagnosis of a cancer related death was made autopsy confirmed this in only 75% of the cases. Moreover, breast cancer was raised as a possible cause of death in a substantial proportion of patients who were subsequently found to have died from unrelated causes. With hindsight some of these cases may have been treatable had it been possible to form an accurate clinical diagnosis. In general there seems to be a clinical tendency to over-diagnose breast cancer as a cause of death when a patient has been previously treated for this disease. To a much lesser extent this study has also shown that the occasional breast cancer death may be missed clinically.

These findings are important, as clinical research commonly uses mortality statistics in the assessment of prognostic factors and therapeutic regimes. These studies often require large numbers of patients to achieve statistically significant results and this number could possibly be reduced if accurate mortality statistics based on autopsy data were available.

An unexpected finding of this study is that only 29% of the patients with treated breast cancer actually died as a

**Table IV** Pattern of metastases in 85 patients (number of cases microscopic in parentheses)

| | *Number* | | *Percentage* | *Previously reported* (%)[a] |
|---|---|---|---|---|
| Heart | 3 | (3) | 4 | 5–13 |
| Pericardium | 7 | (1) | 8 | 19–35 |
| Heart + pericardium | 9 | (3) | 10 | 22 |
| Lungs | 18 | (11) | 21 | 57–77 |
| Lungs + pleura | 23 | (7) | 27 | 75 |
| Gastrointestinal tract | 3 | | 4 | 14–30 |
| Liver | 19 | | 22 | 50–71 |
| Peritoneum | 4 | (1) | 5 | 13–33 |
| Pancreas | 1 | | 1 | 11–17 |
| Kidneys | 2 | (1) | 2 | 11–27 |
| Adrenals | 4 | (2) | 5 | 30–54 |
| Spleen | 1 | | 1 | 11–18 |
| Ovaries | 1 | (1) | 1 | 12–23 |
| Regional LN | 12 | | 14 | 50–76 |
| General LN | 10 | (1) | 12 | |
| Bone | 17 | | 20 | 49–74 |
| Muscle (not local) | 1 | | 1 | |
| Brain | 4 | (1) | 5 | 10–36 |
| Meninges | 2 | | 2 | 5–39 |
| Brain + meninges | 5 | (1) | 6 | 7–31 |

[a]Abrams *et al.*, 1950; Meissner & Warren, 1971; Sproul, 1971; Cifuentes & Pickren, 1979; Cho & Choi, 1980; Hagemeister *et al.*, 1980; Amer, 1982.

direct result of the disease. This is contrary to a figure of 92% found by Hagemeister *et al.* (1980) and 84% by Cho & Choi (1980). However, the modes of death (carcinomatosis, infection, haemorrhage) in the patients who died as a consequence of breast cancer are similar in all the studies. This discrepancy in the proportion of patients dying of breast cancer may result from differences in the nature of breast cancer or the effectiveness of treatment in the United Kingdom when compared with the United States of America. More likely, however, is the possibility that the figures may be biased and reflect the pattern of autopsy request by clinicians in different centres rather than the true mortality experienced by breast cancer patients in general. It is of interest that the overall survival figures for our 85 patients is comparable with those published in clinical studies (Gazet, 1981).

These points may also account for the fact that the incidence of metastases observed in this study are substantially less than those previously reported (Abrams *et al.*, 1950; Meissner & Warren, 1971; Sproul, 1971; Cifuentes & Pickren, 1979; Cho & Choi, 1980; Amer, 1982). However, it must be stated that none of the previous papers indicated what proportion of the metastases were microscopic. Differences in tissue sampling for microscopy between centres could significantly alter these results. Micrometastases, while important in long-term patient prognosis, are generally of no significance in the immediate cause of death.

The list of diseases leading to death in those patients clinically suspected of dying of breast cancer but dying of independent causes is similar to the list in all non-breast cancer deaths. This suggests that no particular disease is likely to mimic breast cancer. However, one particularly striking feature is the high incidence of a second primary neoplasm. The significance of this is uncertain but it may be related to the increasing age of the population with secondary immunodeficiency, genetic factors or modes of treatment.

In conclusion this study has highlighted the need for accurate mortality data in clinical research, treatment and public health planning and emphasises the need to improve autopsy rates, which have been falling in this country for some time. A detailed prospective autopsy study of patients with breast carcinoma is currently in progress in Tayside. Information of this type will become particularly important when mammographic breast screening is introduced in the near future.

We would like to thank Professor J.S. Beck for his support with this study.

**References**

ABRAMS, H.L., SPIRO, R. & GOLDSTEIN, N. (1950). Metastases in carcinomas: analysis of 1,000 autopsied cases. *Cancer*, **3**, 74.

AMER, M.H. (1982). Chemotherapy and pattern of metastases in breast cancer patients. *J. Surg. Oncol.*, **19**, 101.

CAMERON, H.M. & McGOOGAN, E. (1981). A. prospective study of 1152 hospital autopsies: I. Inaccuracies in death certification. *J. Pathol.*, **133**, 273.

CHO, S.Y. & CHOI, H.Y. (1980). Causes of death and metastatic patterns in patients with mammary cancer: ten year autopsy. *Am. J. Clin. Pathol.*, **73**, 232.

CIFUENTES, N. & PICKREN, J.K. (1979). Metastases from carcinoma of mammary gland: an autopsy study. *J. Surg. Oncol.*, **11**, 193.

EDITORIAL (1966). Accuracy of death certificates. *Lancet*, **ii**, 1349.

GAZET, J.-C. (1981). Surgical management of breast cancer. In *Breast Cancer Management*, Coombes, R.C., Powles, T.J., Ford, H.T. & Gazet, J.-C. (eds) p. 104. Academic Press: London.

HAGEMEISTER, F.B., BUZDAR, A.U., LUNA, M.A. & BLUMENSCHEIN, G.R. (1980). Causes of death in breast cancer: a clinicopathologic study. *Cancer*, **46**, 162.

MEISSNER, W.A. & WARREN, S. (1971). Sites of metastases at autopsy. In *Pathology, vol. 1*, Anderson, W.A.D. (ed) p. 538. C.V. Mosby: St Louis.

MOULD, R.F. (1983). *Cancer Statistics*, Medical Science Series. Adam Hilger: Bristol.

SPROUL, cited by HAAGENSEN, C.D. (1971). The natural history of breast carcinoma. In *Diseases of the Breast*, 2nd edn., p. 426. W.B. Saunders: Philadelphia.

VIADANA, E., COTTER, R., PICKREN, J.W. & BROSS, I.D.J. (1973). An autopsy study of metastatic sites of breast cancer. *Cancer Res.*, **33**, 179.


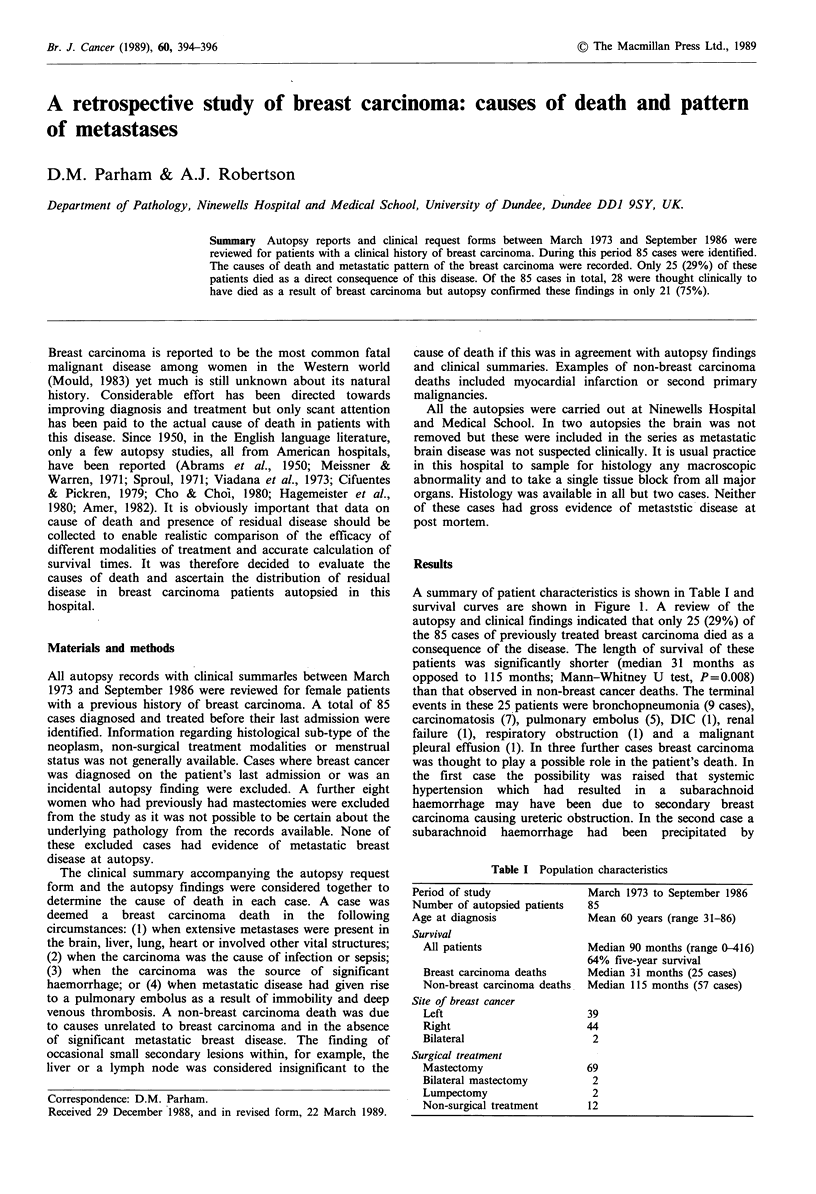

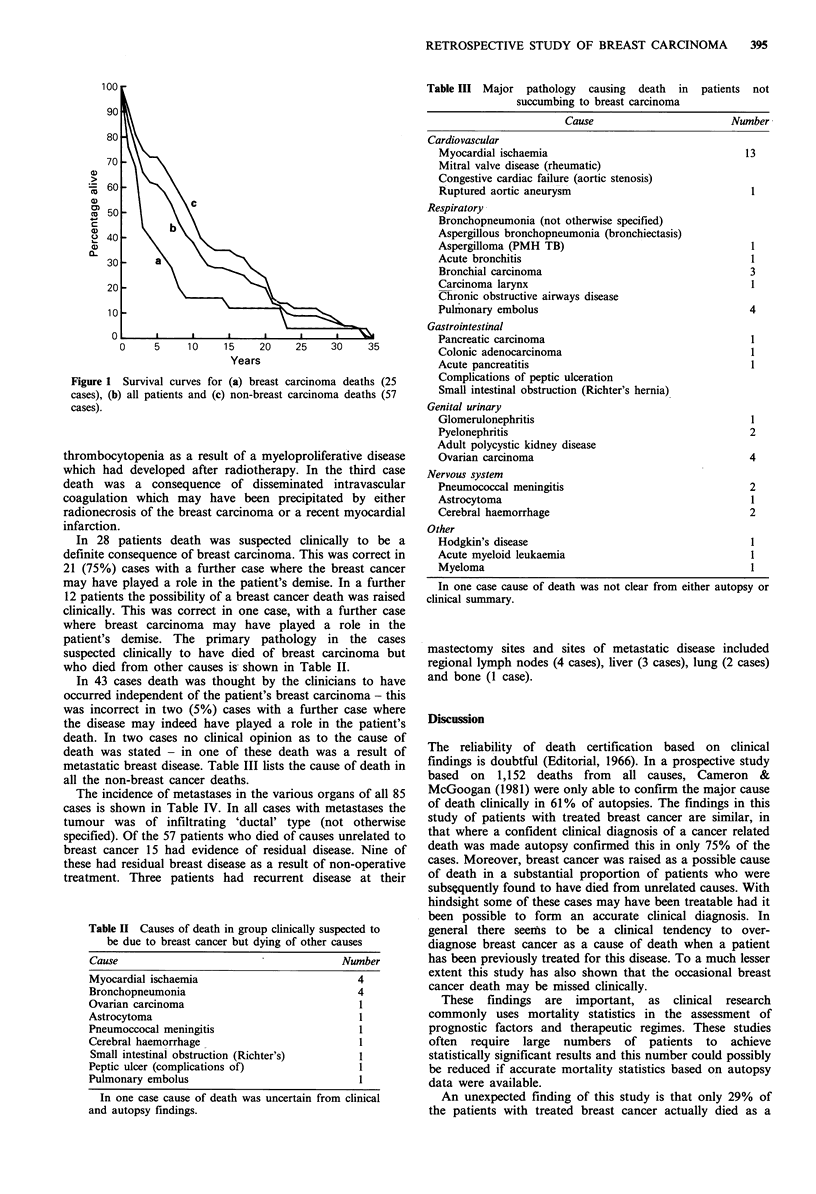

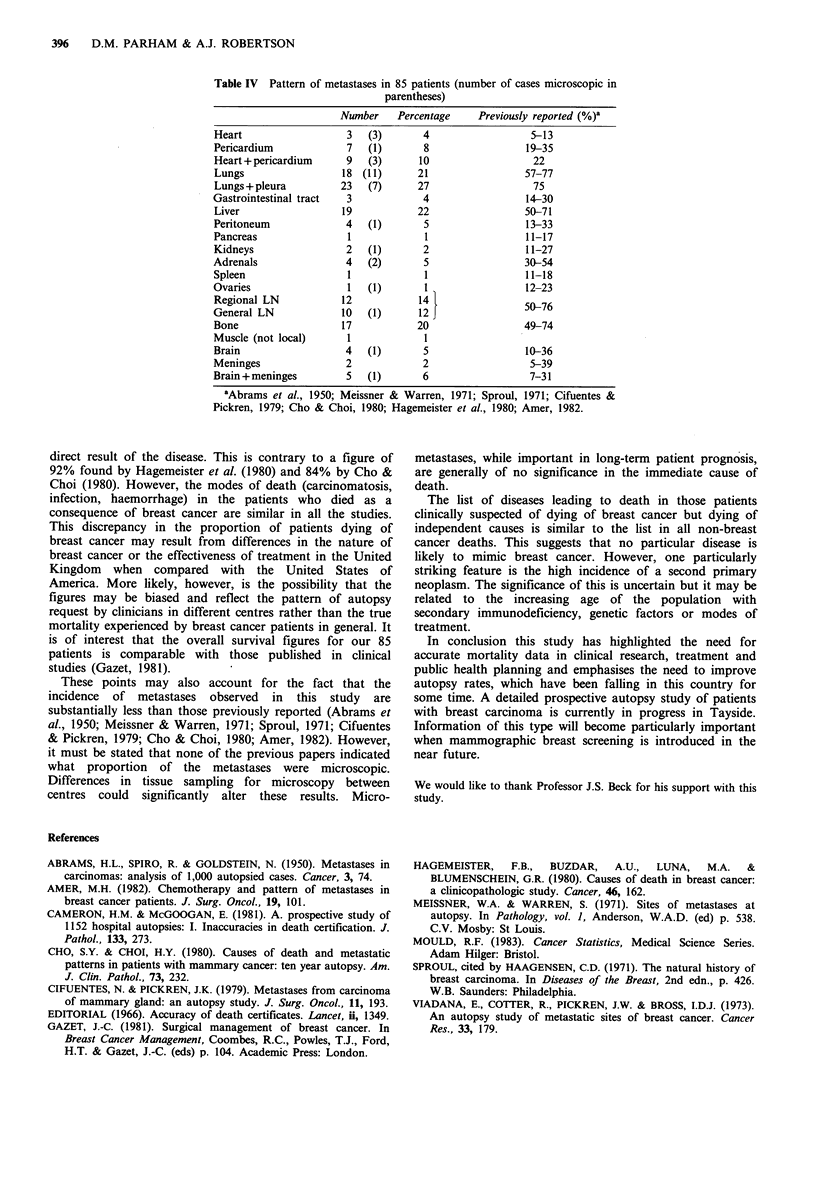


## References

[OCR_00438] ABRAMS H. L., SPIRO R., GOLDSTEIN N. (1950). Metastases in carcinoma; analysis of 1000 autopsied cases.. Cancer.

[OCR_00442] Amer M. H. (1982). Chemotherapy and pattern of metastases in breast cancer patients.. J Surg Oncol.

[OCR_00446] Cameron H. M., McGoogan E. (1981). A prospective study of 1152 hospital autopsies: I. Inaccuracies in death certification.. J Pathol.

[OCR_00451] Cho S. Y., Choi H. Y. (1980). Causes of death and metastatic patterns in patients with mammary cancer. Ten-year autopsy study.. Am J Clin Pathol.

[OCR_00456] Cifuentes N., Pickren J. W. (1979). Metastases from carcinoma of mammary gland: an autopsy study.. J Surg Oncol.

[OCR_00465] Hagemeister F. B., Buzdar A. U., Luna M. A., Blumenschein G. R. (1980). Causes of death in breast cancer: a clinicopathologic study.. Cancer.

[OCR_00484] Viadana E., Cotter R., Pickren J. W., Bross I. D. (1973). An autopsy study of metastatic sites of breast cancer.. Cancer Res.

